# Concentration and health risk assessment of melamine in commercial citrus juices

**DOI:** 10.1016/j.fochx.2025.102254

**Published:** 2025-02-03

**Authors:** Marzieh Rashedinia, Behrouz Akbari-Adergani, Parisa Shavali-gilani, Razieh Noroozi, Mehdi Fathollahi, Parisa Sadighara

**Affiliations:** a Department of Pharmacology and Toxicology, School of Pharmacy, Shiraz University of Medical Sciences, Shiraz, Iran; b Water Safety Research Center, Food and Drug Administration, Ministry of Health and Medical Education, Tehran, Iran; c Department of Environmental Health, Food Safety Division, School of Public Health, Tehran University of Medical Sciences, Tehran, Iran

**Keywords:** Melamine, Fruit juice, Health risk assessment, High performance liquid chromatography

## Abstract

Melamine contamination in food poses significant health risks including kidney stones, renal failure, making it a critical food safety concern. This study investigated the presence of melamine contamination in commercial citrus juices and its exposure through citrus juice. Samples were selected from different packaging. Melamine concentrations in commercial juice samples varied significantly, ranging from 1.732 mg/L in lemon juice to 31 mg/L in orange juice. The highest levels were found in products packaged in cardboard packaging. However, the risk assessment did not identify any risk for different ages. But, these findings highlight the need to monitor melamine levels in commercial juices.

## Highlights

• Melamine detected in citrus-based fruit juice samples.
• Melamine was higher in orange juice than lemon juice.
• There is a correlation between the type of packaging and the amount of melamine in fruit juice.

## 1 Introduction

In recent years, public awareness of food safety has significantly increased, particularly due to the contamination of various agricultural products with harmful substances (Noroozi et al., 2022). One such contaminant that has garnered much attention is a heterocyclic aromatic compound with the chemical formula C_3_H_6_N_6_ called melamine. Melamine is a white crystalline powder with moderate solubility in water, and its crystalline structure is mainly made up of nitrogen. Melamine is widely used in the production of various plastics and resins, coatings, industrial filters, adhesives, and specific types of containers. Melamine contamination in food can occur through packaging materials, kitchen utensils, adhesives, coatings, pesticides and herbicides, sanitizers and disinfectants, and water contamination, posing significant food safety risks (Alizadeh et al., 2023). It leads to severe health risks, including kidney stones and renal failure. Melamine is absorbed in the digestive tract and form crystals in the kidneys, which lead to the formation of kidney stones(Rovina & Siddiquee, 2015). This compound has also caused male reproductive toxicity in animals(Chang et al., 2018). Following toxicokinetic studies in rats, it has been observed that oral absorption of melamine in the gastrointestinal tract is very high, reaching approximately 73 to 98 %(Charron et al., 2024). 90 % of ingested melamine is excreted in the urine within the first 24 h (Jong Hyuk Kim, 2023; J. H. Kim et al., 2019). Most melamine is excreted without being metabolized in mammals, although it may be converted to cyanuric acid by intestinal bacteria(Tebby, Brochot, Dorne, & Beaudouin, 2019).

Melamine has been categorized by the International Agency for Research on Cancer (IARC) as “possibly carcinogenic to humans” (Group 2B)(Muñoz et al., 2023). Due to these risks, global regulatory agencies have established strict limits on melamine levels in food products. The Codex Alimentarius Commission, for instance, has determined a maximum melamine level of 2.5 mg/kg in powdered infant formula and 1 mg/kg in liquid milk products(Strashnov, Karunarathna, Fernando, Dissanayake, & Binduhewa, 2021). Furthermore, World Health Organization (WHO) suggests a tolerable daily intake (TDI) of 0.2 mg/kg of body weight for melamine (Filazi, Sireli, Ekici, Can, & Karagoz, 2012; Hu et al., 2019).

Melamine was added to baby food as an adulterant in 2008. But, melamine is currently being detected in various foods that are clearly unrelated to the issue of adulteration(Chain, E. P. o. C. i. t. F., EFSA Panel on Food Contact Materials, E., Flavourings, and Aids, P, 2010). Citrus fruits and their juices are celebrated for their high nutritional value, primarily because of their substantial vitamin C content. Additionally, they are rich in bioactive compounds like carotenoids, flavonoids, and provide essential vitamins including B vitamins, thiamine, riboflavin, niacin, folic acid, inositol, biotin, and choline(Kowalska, Konopska, Feszterová, Zbikowska, & Kowalska, 2023). Despite their nutritional benefits, citrus juices are not immune to contamination, and potential contamination of those with melamine particularly due to their widespread consumption across vulnerable populations such as children and the elderly is a significant public health concern (Zhu & Kannan, 2019). Extensive investigations have been carried out regarding melamine contamination in dairy products (Alizadeh et al., 2023; Chu et al., 2024; Filazi et al., 2012). However, there is a significant lack of studies concentrating on non-dairy food items like fruit juices. This gap underscores the need for more studies to identify melamine in various food products including citrus juices. Citrus juice extraction is usually done mechanically using presses. It is possible that the structure of these presses is made of melamine. Given that citrus fruits have thicker skins than other fruits, more mechanical pressure is required. Therefore, the possibility of melamine migrating into citrus fruit juice is higher than that of other fruits. Therefore, this research investigates the occurrence of melamine in commercial citrus juices and estimates dietary exposure among different demographic groups to assess potential health risks.

## 2 Materials and methods

### 2.1 Chemicals and reagents

Crystalline melamine and Graphite carbon black (GCB) were purchased from Sigma Aldrich. HPLC-grade solvents such as methanol, acetonitrile, and formaldehyde were obtained from Merck (Darmstadt, Germany). The water used in each experiment was produced by a water purification system from Millipore in the USA.

### 2.2 Preparation of standard solutions, validation parameters, and spiking process

The melamine stock solution (100 mg/ml) was prepared by dissolving 500 mg of melamine standard to 5 ml HPLC grade water and kept frozen (−20 °C) in an amber vial until samples analysis. The proper dilutions of the stock solution in the water (1:9, *v*/v), were utilized to prepare the working standard solutions, with ranging concentrations (0.05 to 60 mg/ml), then were kept at 4 °C. Quantification of samples was carried out using calibration curves prepared with standards in the solvent, containing the internal standard to provide a relative response. Recovery experiments involved spiking samples at levels of 1 and 2.5 mg/L (Table 1). Spiking of beverage samples to simulate the possible highest level of melamine contamination, and a recovery experiment was performed by adding 1 mL of the 10 or 25 mg/L standard solution to 10 mL of blank beverage (1 and 2.5 mg/ L, respectively) and spiked samples were equilibrated for 1 h before extraction (Ibánez, Sancho, & Hernández, 2009). Furthermore, LOD (Limit of Detection) and LOQ (Limit of Quantitation) amount were 0.017 and 0.052 μg/mL.

**Table 1:** Recovery values of melamine spiked in beverages at two levels.

Beverage Sample	Recovery factor	Recovery Level	
Orange Juice	Spiked level (mg/L)	1.00 mg/L	2.50 mg/L
	Mean Found value (mg/L)	0.98	2.43
	Mean Recovery (%) ± RSD (*n* = 3)	98.01 ± 0.33	97.20 ± 0.29
Citrus Juice	Spiked level (mg/L)	1.00 mg/L	2.50 mg/L
	Mean Found value (mg/L)	0.97	2.41
	Mean Recovery (%) ± RSD (n = 3)	97.05 ± 0.28	96.40 ± 0.22

### 2.3 Sampling

Commercial juice samples were randomly selected from different brands and packaging, focusing on packaging materials. Five different brands were selected. The selected samples were usually filled in a similar manner in 280 ml containers for individual consumption. The samples had higher consumption and a higher market share. In order to maintain confidentiality and avoid any bias in the test results, the brand names were not mentioned and the samples were coded alphabetically. The packaging materials for fruit juices were glass packaging, cans, polyethylene terephthalates (PET), and cardboard. All samples were stored at 4 °C until analysis.

### 2.4 Sample extraction and analysis

Sample preparation for melamine analysis was done according to the Ibáñez et al.'s method with a little change(Ibánez et al., 2009). To analyze the beverages, 5.0 mL of each sample were transferred to centrifuge tubes (50 mL) followed by adding 10 mL of acetonitrile and 15 g of Graphitized Carbon Black (GCB). Liquid samples were shaken for 40 min, followed by centrifugation at 4500 rpm for 15 min. Afterward, the supernatant was separated with a syringe and purified after passing through the filter. The gathered solvent was evaporated to dryness under a gentle stream of nitrogen, reconstituted with 200 μL of mobile phase solution, filtered through nylon syringe filter directly into a glass vial, and saved at −18 °C until analysis. Eventually, 50 μl elution was injected into the HPLC. The quantification of melamine concentrations was conducted in triplicate utilizing a high-performance liquid chromatography (HPLC) system, which was fitted with a UV detector at 254 nm. The mobile phase consisted of acetonitrile and water (90:10) with a flow rate of 1.0 ml/min.

### 2.5 Assessment of human health risks

For health risk assessment, chronic daily intake of melamine in citrus juice consumption assumptions were used to survey long-term health risks to consumers. CDI of melamine in adults and children consumers was calculated using below equation:

(1) $$\mathrm{CDI}=\frac{C\times \mathrm{IR}\times \mathrm{ED}\times \mathrm{EF}}{\mathrm{BW}\times \mathrm{AT}}$$

CDI (chronic daily intake)(mg/kg/day).

C or concentration of melamine in fruit juice samples (mg/kg), IR or ingestion rate that per capita consumption of fruit juice is considered, which is considered to be 2 g per day based on official reports. ED (exposure duration) is considered to be 70 years and EF (exposure frequency) is considered to be 365 days per year and body weight is considered to be 70 kg in adults and 15 kg for children.

Health risk indices (HI) were derived by dividing the CDI by the, ADI (mg/kg/day) using the following Eq. 2:

(2) HI=CDI/ADI

When the HI > 1; it is considered a risk to the concerned consumers. While the HI < 1 means it is considered as acceptable to the concerned consumers(Zhu & Kannan, 2019).

### 2.6 Statistical analysis

The data was analyzed using SPSS software (version 22.0, USA) and the results were reported as mean ± standard deviation. The variation in melamine levels among different packaging types was evaluated using a one-way analysis of variance (ANOVA) and Duncan's post-hoc test to identify significant differences between groups. A significance level of *p* < 0.05 was considered significant for all experimental data.

## 3 Results

### 3.1 The concentration and health risk assessment

Table 2 shows the amount of melamine by type of fruit juice. The amount of melamine was higher in orange juice than in lemon juice. A significant difference was observed between the amounts of melamine depending on the type of juice (*p*=0.01).

**Table 2:** Results of melamine concentration and risk assessment in fruit juice samples.

Type of juice	Min-Max(mg/L)	Mean ± SD(mg/L)	HQ(adult)	HQ(children)
Orange	9.2–31	15.9 ± 1.9	0.002	0.01
Lemon	1.7–2.1	1.9 ± 0.2	0.0002	0.001

**Fig. 1:**
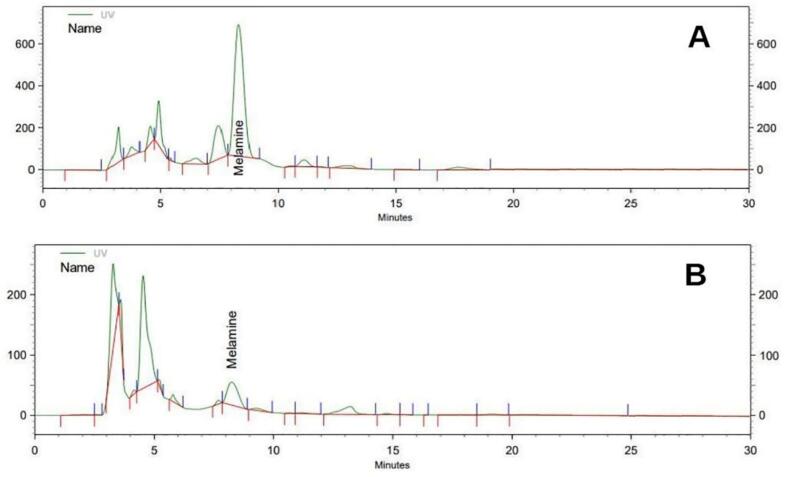
HPLC chromatograms for determination of melamine in orange sample (A) and lemon sample (B).

Fig. 1 shows the peaks obtained from the amount of melamine in two samples of orange juice (A) and lemon juice (B).

**Fig. 2:**
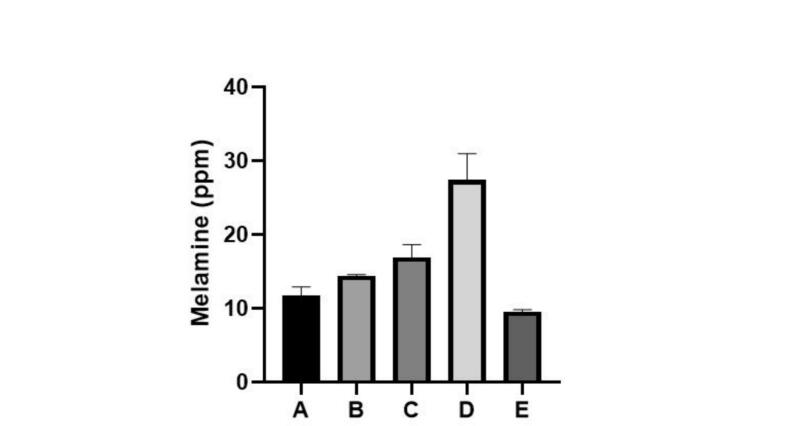
Melamine content by brand of orange juice.

The Fig. 2 shows the results of melamine concentrations (mg/L) in commercial orange juice samples from different brands. A significant difference was observed between Brand D and other brands. No significant differences were observed between other brands.

### 3.2 The concentration of melamine by the package type

**Fig. 3:**
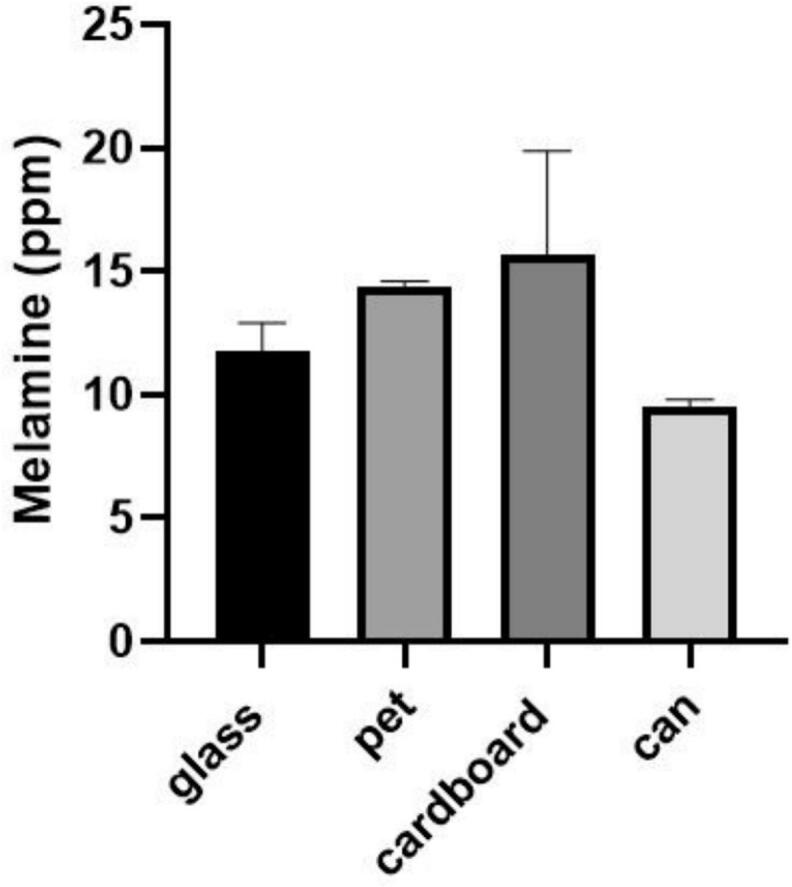
Melamine content by packaging type.

Given that the type of packaging also affects melamine content, a study was also conducted on the packaging material (Fig. 3). The highest melamine content was found in cardboard packaging.

## 4 Discussion

Both liquid chromatography and gas chromatography-based methods are well developed for the determination of melamine in foods (Alizadeh Sani et al., 2023; Joshi et al., 2023). In this study, HPLC was used for evaluating of melamine in a variety of citrus-based juices. Fruits and beverages are one source of exposure to melamine. A study found a correlation between urinary melamine levels and certain food items. One of these foods was fruits(Shi et al., 2020). Furthermore, a study in Thailand was conducted to determine the level of melamine exposure in various foods. One of the foods that was considered a source of melamine exposure in this regard was beverages(Lapviboonsuk & Lapviboonsuk, 2020).

The complex supply chains and production processes in commercial citrus juice manufacturing can cause contaminants such as melamine through several pathways. The analysis of this study showed that the highest and lowest melamine concentrations recorded were 31 mg/L for orange juice and 1.732 mg/L for lemon juice, respectively, indicating a significant difference in melamine content based on the type of juice(Table 2). This difference is probably due to the thicker skin of oranges compared to lemons. Fruit juice extraction is done mechanically. Plastic tools are usually used for this. It is possible that these plastic equipment contain melamine. It generally requires more mechanical force to extract juice from oranges than from lemons. This may result in more melamine migrating from melamine-containing equipment into the oranges during juicing. In addition, the rate of melamine migration from worn-out equipment is also higher(Mannoni et al., 2017).

Another important reason for the presence of melamine in fruits and vegetables is the use of the insecticide cyromazine (Melough, Foster, Fretts, & Sathyanarayana, 2020; Shi et al., 2020). The use of pesticides has increased in recent years(Shirani et al., 2024). Melamine is the main breakdown product of cyromazine(Guo et al., 2020).

In addition, it is possible that the plant takes up melamine from water and soil. Due to the widespread use of melamine, this compound is found in a wide range of different environments(J. Li et al., 2022). One of the factors that cause soil contamination with melamine is fertilizers. The study examined melamine in soils and fertilizers used in agriculture(Zhu, Wang, Sun, & Kannan, 2019). Melamine was detected in both soil and fertilizers. Its concentration in fertilizers was 3 to 4 times higher than in soils. Among fertilizers, high concentrations of melamine and cyanuric acid were observed in nitrogen fertilizers(Zhu, Wang, et al., 2019). Melamine has been detected in drinking water and wastewater (J. Li et al., 2022; Zhu, Halden, & Kannan, 2019). Melamine is not well removed from water in conventional wastewater treatment plants due to its low biodegradability(Piai, van der Wal, Boelee, & Langenhoff, 2021). In one study, significant amounts of melamine were observed in lake sediments. The lake was located in a heavily industrialized area(Zhu, Lee, Moon, & Kannan, 2019). Also, in another study, you identified melamine in the water of a river(Dsikowitzky & Schwarzbauer, 2015). Usually, there is melamine contamination following industrial activity and the discharge of wastewater and sewage into the water.

In addition, there is also the possibility of melamine migrating through packaging. Melamine is a chemical compound widely used in packaging(Ziarati et al., 2018). One source of exposure to melamine is from the migration of this compound through packaging(Lu et al., 2017). Significant amounts of melamine were found in cardboard packaging (Fig. 3). Paper and cardboard-based packaging has always been popular in the food industry due to its low cost and bio renewability(Z. Li & Rabnawaz, 2018). Melamine is used in the construction of cardboard and paper (H. S. Kim et al., 2021; Sarafraz Yazdi, Raouf Yazdinezhad, & Heidari, 2015; Wang et al., 2023). This compound is also used in paper coatings(Biabani, Nezhadali, Nakhaei, & Nakhaei, 2020). Paper cups are coated with melamine, which is FDA approved for food contact paper(Li & Rabnawaz, 2018). Therefore, it is likely that some of the melamine present in the cardboard-packaged samples is due to migration from the packaging. Similar results to the present study were observed in the study by Mazaheri et al. In this study, the amount of melamine was measured in milk samples with cardboard, plastic and nylon packaging. The highest amount of melamine was observed in the samples with cardboard packaging(Ghanati et al., 2023). After cardboard packaging, the highest amount was observed in plastic packaging. Melamine is also used in the manufacture of plastics (Jong Hyuk Kim, 2023; Taşci, Çubuk, Yetimoğlu, & Kahraman, 2022). The release of melamine from plastic packaging is still considered a risk(Ebner et al., 2020). The lowest amount of melamine was observed in can packaging (Fig. 3). These packages are usually made of metals such as aluminum (Chuchot & Thanakijkasem, 2024; Önen, 2022). The packaging material of the fruit juice cans in this study was metallic. Therefore, melamine is not present in the structure of this type of packaging.

## 5 Conclusion

In this study, two types of citrus fruits, lemon juice and orange juice, were selected to determine the amount of melamine. The amount of melamine in orange juice was higher than in lemon juice. Also, different amounts of melamine were seen in different brands. However, by assessing the risk, no risk was calculated for the two age groups of adults and children. Also, in this study, the role of the effect of the type of packaging on the amount of melamine in fruit juices was considered. The highest amount was observed in cardboard packaging. Given that cardboard and paper are renewable and their price is low, the use of this type of packaging is widespread. Therefore, their safety should be considered in further research.

## Ethical approval

Not applicable.

## Consent to participate

Not Applicable.

## Consent for publication

Not Applicable.

## Funding

This study was financially supported by 10.13039/501100004320Shiraz University of Medical Sciences (Grant No.: 29108, Ethics code: IR.SUMS.REC.1402.448).

## CRediT authorship contribution statement

**Marzieh Rashedinia:** Methodology. **Behrouz Akbari-Adergani:** Investigation. **Parisa Shavali-gilani:** Methodology. **Razieh Noroozi:** Methodology. **Mehdi Fathollahi:** Investigation. **Parisa Sadighara:** Writing – review & editing, Writing – original draft.

## Declaration of competing interest

The authors declare that they have no known competing financial interests or personal relationships that could have appeared to influence the work reported in this paper.

## Data Availability

The data that has been used is confidential.
